# BCNU (Bis-chloroethylnitrosourea, Carmustine) Toxicity Presented as a Large Pleural Effusion 60 Days Post Autologous Stem Cell Transplant for Non-Hodgkin Lymphoma

**DOI:** 10.7759/cureus.4052

**Published:** 2019-02-12

**Authors:** Shelby N Dvorak, Peter C Kurniali

**Affiliations:** 1 Family Medicine, The Christ Hospital / University of Cincinnati Family Medicine Residency, Cincinnati, USA; 2 Oncology, Sanford Health / University of North Dakota School of Medicine and Health Sciences, Bismarck, USA

**Keywords:** bcnu, non-hodgkin lymphoma, pleural effusion

## Abstract

Non-Hodgkin lymphoma is a commonly encountered malignancy. Treatment for advanced stages commonly consists of chemotherapy followed by autologous stem cell transplant. BCNU (bis-chloroethylnitrosourea, carmustine) is frequently used as part of the conditioning regimen for autologous stem cell transplant. BCNU is well known to cause pulmonary toxicity, but it is uncommon for pulmonary toxicity to present as a pleural effusion. In our case, a patient with non-Hodgkin lymphoma, particularly mantle cell lymphoma, was given BCNU prior to autologous stem cell transplant. The BCNU resulted in the patient developing large bilateral pleural effusions 60 days post transplant. Knowledge of this potential complication following BCNU use as well as proper treatment can help patients avoid excessive medical visits and tests.

## Introduction

Recent data from the National Cancer Institute estimates over 72,000 new cases of non-Hodgkin lymphoma occur annually [1]. This comprises about 4% of all new cancer cases and places it among the 10 most common malignancies [1-2]. Mantle cell lymphoma is a type of non-Hodgkin lymphoma and comprises approximately 6% of cases of non-Hodgkin lymphoma [3-4]. Males develop mantle cell lymphoma two to three times more frequently than females [3-4]. The median age of diagnosis is 65 years, with the median overall survival exceeding five years [3-4]. Whites are affected slightly more frequently than blacks and Asians [2].

Patients with mantle cell lymphoma can present with fever, night sweats, or weight loss, but many are asymptomatic [3]. The involvement of bone marrow, blood, and the gastrointestinal tract is commonly seen at the time of diagnosis [4]. About one-quarter of patients experience gastrointestinal symptoms, such as abdominal pain, bloating, diarrhea, small-bowel obstruction, and hematochezia [3]. Up to 90% of patients may have asymptomatic gastrointestinal involvement, making endoscopy and random biopsies important [3].

There are a variety of treatment options for mantle cell lymphoma based on the stage of disease and certain patient factors. Treatment of advanced mantle cell lymphoma commonly consists of intensive chemotherapy or intensive chemotherapy plus autologous stem cell transplant [3]. Prior to stem cell transplant, patients often undergo BEAM conditioning, which consists of BCNU (bis-chloroethylnitrosourea, carmustine), etoposide, Ara-C (cytarabine), and melphalan. In addition to non-Hodgkin lymphoma, BCNU has also been used in conditioning protocols for Hodgkin lymphoma, gliomas, breast cancer, and multiple myeloma [5]. BCNU has been known to cause adverse effects, such as pulmonary toxicity, sinusoidal obstruction syndrome, hemorrhagic cystitis, and nephrotoxicity [6]. One of the most common of these is pulmonary toxicity [5]. The most common reports of pulmonary toxicity involve cough, dyspnea, reduced lung diffusion capacity, or chronic interstitial fibrosis [5]. There are rare reports of pulmonary toxicity presenting as pleural effusion [7-8]. Although pulmonary toxicity has been reported to occur as much as 17 years after starting BCNU, symptoms most often appear within one year [5]. Damage to the glutathione system appears to be the cause of these adverse pulmonary effects [5].

In this case report, we present a patient with mantle cell lymphoma, who received BCNU as part of his conditioning for autologous stem cell transplant. Approximately two months after receiving BCNU, he presented with dyspnea and was found to have bilateral pleural effusions.

## Case presentation

**Figure 1:**
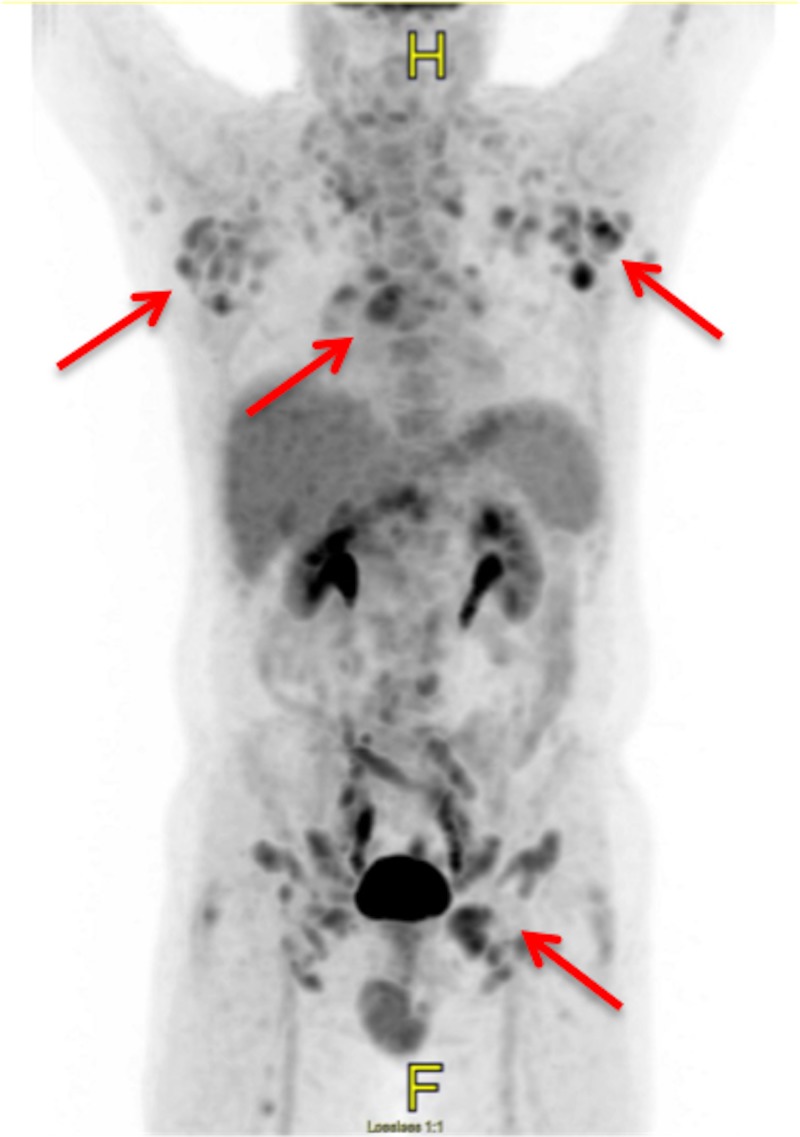
Initial PET/CT PET/CT scan at diagnosis showing extensive metabolically active lymphadenopathy (arrows). PET/CT: positron emission tomography/computed tomography

A 63-year-old male was found to have a polypoid colonic lesion during screening colonoscopy in January 2016. The polyp was biopsied and was found to have involvement by mantle cell lymphoma. Bone marrow aspirate and biopsy showed low-level involvement by mantle cell lymphoma. During the few months prior to diagnosis, the patient had been experiencing fatigue, loss of energy, and subjective fever. After his diagnosis, he began experiencing abdominal bloating and insomnia. The insomnia is believed to be due to anxiety about the diagnosis. He denied night sweats or weight loss. Original laboratory tests showed a normal complete blood count (CBC) with differential, beta-2 microglobulin, lactate dehydrogenase (LDH), and uric acid, and an unremarkable comprehensive metabolic panel (CMP). On computed tomography (CT) of the chest, abdomen, and pelvis, lymphadenopathy was found both above and below the diaphragm, with the largest lymph node being in the left groin, measuring up to 2.5 cm in the short axis. There was no splenomegaly. A positron emission tomography/computed tomography (PET/CT) scan showed metabolically active adenopathy in the supraclavicular, subpectoral, and axillary regions, as well as the middle mediastinum, subcarinal space, superficial and deep inguinal chains, and periaortic region of the lower abdomen (Figure 1). Based on the diagnostic testing, the mantle cell lymphoma was classified as stage IV. The patient did not undergo next-generation sequencing, so p53 mutation status was not available. Treatment was started in February 2016.

**Figure 2:**
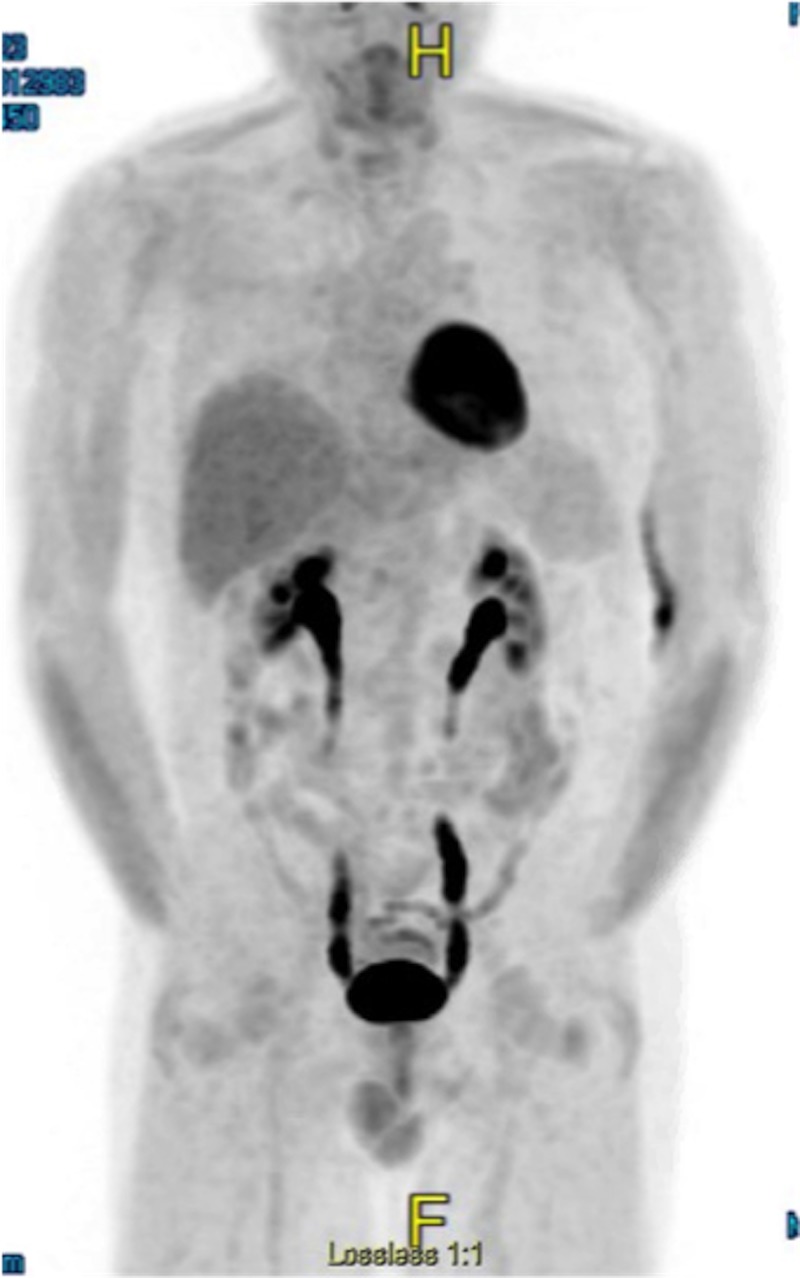
Post-treatment PET/CT PET/CT scan after treatment showing resolution of previously seen lymphadenopathy. PET/CT: positron emission tomography/computed tomography

The patient was referred to a specialty hospital to determine the optimal treatment regimen. The decision was made to proceed with the Nordic protocol, consisting of Maxi-CHOP (cyclophosphamide, doxorubicin, vincristine, and prednisolone), high-dose cytarabine, and rituximab. In addition, the patient was given pegfilgrastim on the second day of each chemotherapy cycle. After completing the six cycles of chemotherapy, the patient was found to be in complete remission (Figure 2). Shortly after, the patient returned to the specialty hospital where he underwent BEAM conditioning, followed by autologous stem cell transplant. He also received filgrastim and plerixafor during this time. The patient was placed on trimethoprim-sulfamethoxazole and penicillin V for post-transplant prophylaxis. He remained at the specialty hospital for several weeks following the transplant, due to complications, including neutropenic fever, orthostatic hypotension, generalized weakness, and poor engraftment, requiring multiple filgrastim injections.

**Figure 3:**
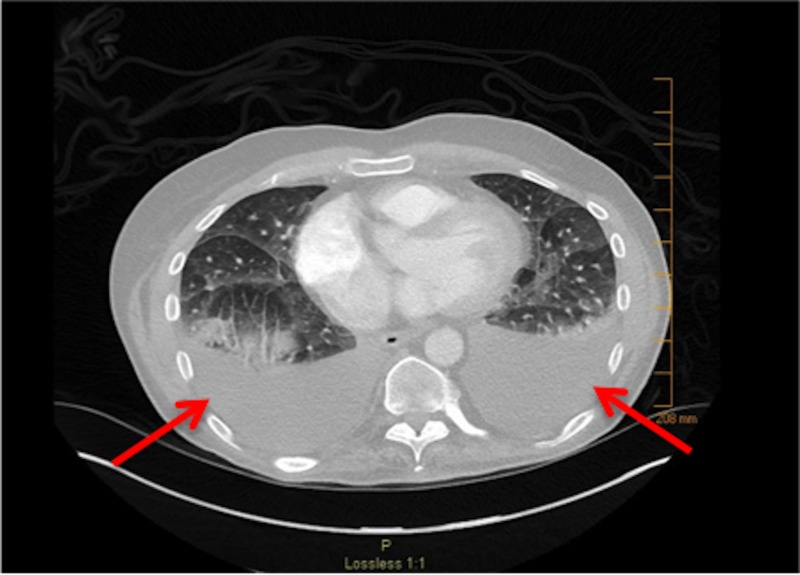
Bilateral pleural effusions CT scan showing bilateral pleural effusions (arrows). CT: computed tomography

**Figure 4:**
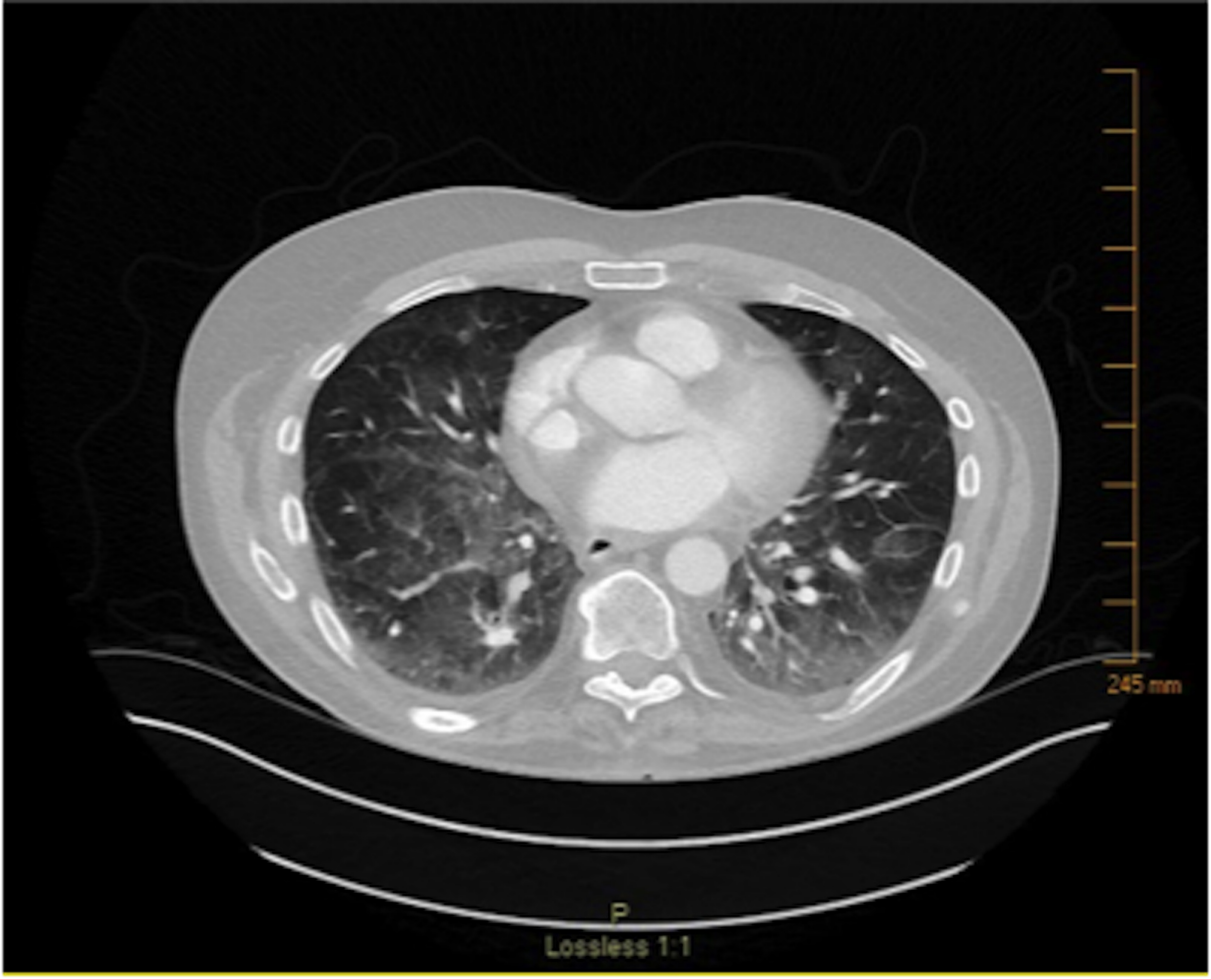
CT after course of steroids CT scan after steroid treatment, showing resolution of previously seen bilateral pleural effusions. CT: computed tomography

Approximately two months after autologous stem cell transplant, the patient presented to the emergency department with dyspnea. He was admitted to the hospital and a CT angiography (CTA) was performed. The CTA was negative for pulmonary embolism but did show large bilateral pleural effusions (Figure 3). Analysis of the pleural fluid using Light's criteria revealed a transudative effusion. Thoracentesis and bronchoscopy did not show any evidence of infection. Doppler of the abdomen was negative for portal vein thrombosis and hepatic vein thrombosis. Two-dimensional echocardiogram revealed a normal left ventricular ejection fraction of 60%-65%, which was unchanged from the patient's baseline echocardiogram. The patient had a remote history of tobacco use of about one pack of cigarettes per day for four years but had not used any tobacco products in over 40 years. He had no history of lung disease. The patient was given two doses of methylprednisolone 125 mg intravenous (IV) eight hours apart. The next day, he was started on prednisone 1 mg/kg of body weight for 10 days and then tapered by 5 mg every two days. The patient’s symptoms completely resolved following steroid administration (Figure 4). The bilateral pleural effusions experienced by the patient are thought to be due to BCNU-related lung toxicity.

## Discussion

Although pulmonary toxicity is a well-known adverse effect of BCNU, certain presentations of pulmonary toxicity are more likely to be seen than others. In general, drug-induced parenchymal disease is far more common than drug-induced pleural disease [9]. We were able to find two previously reported episodes of pleural effusion following BCNU administration in our literature search. A 1998 study by Wilczynski et al. looked at the occurrence of pulmonary toxicity in 45 women with breast cancer undergoing treatment with high-dose chemotherapy and autologous bone marrow transplant [8]. BCNU was part of the treatment regimen for all 45 women. The women were followed for up to 126 weeks. During that time, 26 women developed symptoms of pulmonary toxicity, requiring steroids. Another 18 women had significant changes on pulmonary functions tests but remained asymptomatic. Of these 18, three had changes severe enough to require treatment with steroids. In total, 44 women developed some form of pulmonary toxicity during the study, but only one was found to have pleural effusion on CT scan.

Another report from Krishnan et al. in 2008 describes a 31-year-old female with anaplastic large T-cell non-Hodgkin lymphoma, who presented with bilateral upper lobe pulmonary infiltrates, bilateral pleural effusions, and a large pericardial effusion after receiving BCNU during conditioning for her autologous stem cell transplantation [7]. Her symptoms occurred 35 days post stem cell transplant. The infiltrates and effusions completely resolved with prednisone treatment. Ninety-nine days after transplant, she experienced a reoccurrence of similar radiological findings while taking maintenance prednisone. Following treatment with prednisone, she again experienced complete resolution of the infiltrates and effusions.

While a pleural effusion may not be the most common presentation of pulmonary toxicity caused by BCNU, it is important to know it can occur. If a pleural effusion does develop following BCNU use, the treatment is usually relatively straightforward. The treatment regimen used for both our patient and the patient presented by Krishnan et al. included prednisone at a dose of 1 mg/kg/day [7]. The patient who developed pleural effusion in the study by Wilczynski et al. was treated with prednisone 60 mg/day for two weeks, as were the other symptomatic patients in the study [8]. Prednisone 1 mg/kg of body weight for 10 days was also used in the study by Alessandrino et al. to treat pulmonary toxicity in 17 patients who received BCNU prior to stem cell transplant for hematological malignancies [5]. Fifteen of the 17 patients had a complete response to the prednisone. None of these patients had pulmonary toxicity present as a pleural effusion. Prednisone is successful at managing a pleural effusion resulting from BCNU use, as well as other presentations of pulmonary toxicity caused by BCNU [10].

## Conclusions

It is important to be aware of the potential for pleural effusions following BCNU administration and to recognize it when it occurs. This, along with understanding a local health care provider can almost always manage the treatment and can prevent unnecessary tests and specialty medical visits. In turn, this helps patients avoid further time, financial constraints, and, often, extra anxiety.
